# Self-Image as the Interface Between Eating Disorders and Gender Dysphoria: A Narrative Review

**DOI:** 10.1192/j.eurpsy.2026.10435

**Published:** 2026-07-31

**Authors:** M. I. O. D. Morais, L. V. D. Araújo, P. F. B. D. Araújo, A. B. D. C. Alves, F. O. Barbosa, B. D. A. A. L. T. D. Melo, E. D. F. Correia, V. L. D. Melo-Neto, L. C. Carneiro, A. F. C. Ximenes, D. B. B. Ferreira

**Affiliations:** 1 Universidade de Ciências da Saúde de Alagoas; 2 Universidade Federal de Alagoas, Maceió, Brazil

## Abstract

**Introduction:**

Impaired self-image plays a pivotal role in both eating disorders (EDs) and gender dysphoria. This association is shaped by stigma, neuroendocrine factors, functional impairment, and interpersonal dynamics. Although literature on EDs in LGBTQIA+ populations is scarce, existing studies consistently demonstrate high prevalence rates, particularly among transgender individuals. The Minority Stress Model posits that sexual and gender minorities are more susceptible to psychiatric disorders, including EDs, due to chronic exposure to stress and trauma.

**Objectives:**

To examine the correlation between eating disorders and gender dysphoria, explore patients’ self-image perception, and highlight the physical, psychological, and social impacts associated with this intersection.

**Methods:**

An integrative literature review was conducted using LILACS, SCIELO, and PubMed databases. The search strategy included the descriptors: “Eating Disorder,” “Gender Dysphoria,” and “Body Image.” Additionally, the terms “Self-esteem” and “Mental Health” were used independently in both English and Portuguese. Boolean operators were not applied. A total of 108 articles were initially retrieved, and 23 full-text articles were selected for final analysis based on exclusion criteria: duplication, publication prior to 2020, and lack of relevance to the study’s objectives.

**Results:**

Findings indicate elevated rates of pathological eating behaviors and disordered eating symptoms among individuals with gender dysphoria. Several studies found that transgender youth are more likely to receive ED diagnoses compared to cisgender youth, although prevalence rates varied. These results suggest a strong association between gender identity incongruence and disordered eating behaviors.

**Conclusions:**

Despite the limited number of studies on EDs within LGBTQIA+ populations, current evidence highlights a disproportionately high prevalence among transgender individuals. The intersection of social stigma, gender dysphoria, and the pursuit of unattainable body ideals increases vulnerability to EDs. These findings underscore the need for integrated and gender-sensitive mental health care approaches that address both psychosocial and identity-specific dimensions.

**Disclosure of Interest:**

None Declared

